# Polar Compounds Isolated from the Leaves of *Albertisia delagoensis* (Menispermaceae)

**DOI:** 10.3390/molecules16119153

**Published:** 2011-11-02

**Authors:** Geoffrey E. Hawkes, Helene de Wet, Jia Li

**Affiliations:** 1 School of Biological and Chemical Sciences, Queen Mary University of London, Mile End Road, London E1 4NS, UK; 2 Department of Botany, University of Zululand, P/Bag X1001, Kwa-Dlangezwa 3880, South Africa; 3 Department of Surgery and Cancer, Imperial College London, South Kensington Campus, London SW7 2AZ, UK

**Keywords:** *Albertisia delagoensis*, traditional medicine, roemrefidine, allantoic acid, HPLC-NMR

## Abstract

Aqueous infusions of the leaves of the shrub *Albertisia delagoensis* (Menispermaceae) are used in South Africa in traditional Zulu medicine to alleviate a variety of symptoms, including fever, and intestinal problems. We report the analysis of such an aqueous extract using the HPLC-NMR technique. A number of polar compounds were identified, including *proto*-quercitol, nicotinic acid, allantoic acid, 3,4-dihydroxy-benzoic acid, phthalic acid and the aporphine alkaloid derivative roemrefidine. Allantoic acid and roemrefidine have been fully characterised by ^1^H- and ^13^C-NMR and mass spectrometry. Earlier reports of antiplasmodial activity of roemrefidine and of *A. delagoensis* extracts are correlated with this study and with the antipyretic properties of neutral aqueous extracts.

## 1. Introduction

*Albertisia delagoensis* N.E. Br. Forman [=*Epinetrum delagoensis*] is a suffrutescent of the Menispermaceae family found in Mozambique and north-eastern KwaZulu-Natal, South Africa. A number of *Albertisia* species are used medicinally throughout tropical and sub-tropical Africa [1], however *A. delagoensis* is the only species found in southern Africa, and the leaves are reportedly used as an antipyretic [2], antidiarrhoeal and antiemetic [3,4] and the roots are used for menstrual pain, sexual performance in men, chest pain and back pain, among other uses [3,4,5]. An early phytochemical report on *A*. *delagoensis* [6] described the isolation of three alkaloids from the acidified ethanolic root bark extracts, and reported by mass spectrometry *m/z* (M^+^) values and molecular formulae for the three compounds. On the basis of the mass spectrometry fragmentation patterns it was suggested that all three belonged to the bis-benzylisoquinoline class of alkaloids, but chemical structures were not suggested. The alkaloids were tested [6,7] for cytotoxicity on continuous cell lines (VERO cells) and found to be highly active. More recently the isolation of three alkaloids, cocsoline, cocsuline and *O*-methylcocsoline from dilute sulphuric acid extracts of the rhizomes of *A. delagoensis*, and an additional two alkaloids, cycleanine and dicentrine, from similar extracts of the leaves was reported [3]. Subsequently De Wet *et al*. [4] reported that methanol extracts of the dried, milled rhizomes and leaves exhibited a high level of antiparasitic activity on the chloroquine-resistant Gambian FCR-3 strain of *Plasmodium falciparum*, but the same extracts showed low level cytotoxicity against Graham cells (transformed human kidney epithelium cells). Crude alkaloidal extracts of *A. Delagoensis* leaves showed a high level of cytotoxicity against selected breast, melanoma and renal cancer cell lines [8]. However the above studies, identifying antiplasmodial activity and cytotoxicity used compounds extracted into acidified aqueous or alcoholic solution, whereas in traditional medicine in southern Africa it is the *neutral *aqueous extracts of roots or leaves that are drunk. In the present study we sought to analyse leaf extracts obtained in this traditional manner, and did not seek to target only alkaloids as had been the case in previous studies.

The crude extract mixture was inspected by the hyphenated HPLC-DAD-NMR method, in an on-flow experiment where the ^1^H-NMR spectrometer acted as a detector identifying components of the mixture which were present in sufficient quantity to merit more detailed structural analysis by 2-D NMR methods. Those selected components were then separated and accumulated in multiple HPLC-DAD runs with the selected eluent collected in loops (the loop storage method). The HPLC-DAD-NMR and HPLC-DAD-loop storage are among an array of powerful hyphenated analytical methods employing NMR for molecular structure determination, and have been recently and extensively reviewed [9,10,11].

## 2. Results and Discussion

The chromatograms (HPLC conditions as for the loop storage method) of the extract showed eight peaks which eluted within the first 25 min, were well resolved and symmetric. In addition there was a broad peak (with tailing) with retention time 28.5 min.

The on-flow HPLC-NMR experiment on the extract showed five components of the mixture gave sufficiently strong ^1^H-NMR spectra that it was considered useful to isolate these components in sufficient quantity to enable 2-D NMR experiments for structural elucidation. These five peaks in the chromatogram corresponded to four of the eight well-resolved peaks (retention times 2.0, 7.0, 13.2 and 16.8 min) and the broad (tailing) peak (retention time 28.5 min) identified in the shorter (40 min) run (HPLC conditions for loop storage). The collection and accumulation of these five fractions in capillary loops is described in Section 3.4 below.

**Figure 1:**
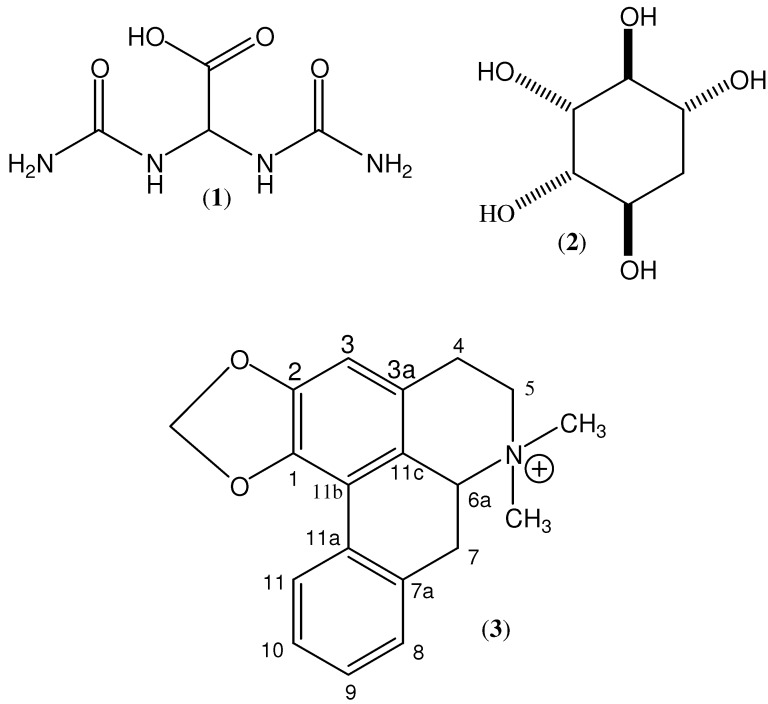
Chemical structures of allantoic acid (**1**), proto-quercitol (**2**), and roemrefidine (**3**).

The ^1^H 1-D NMR spectra of the 2.0 min fraction indicated the presence of a major and two minor components. The 1-D ^13^C spectrum showed six signals, five to higher frequency (71.8, 72.2, 74.5, 75.7 and 78.0 δ), and one at 36.8 δ. The COSY, TOCSY and HSQC spectra are all consistent with this major component being a pentahydroxycyclohexane, and the ^1^H- and ^13^C-NMR data are in agreement with those reported [12] for *proto*-quercitol (**2**, see Figure 1). The two minor components were identified as nicotinic acid [13] and allantoic acid (**1**, see Figure 1).

The ^1^H- and ^13^C-NMR spectra of the 7.0 and 13.2 min fractions showed these to be 3,4-dihydroxy-benzoic acid and phthalic acid, respectively, and these were confirmed by comparison with the spectra of authentic samples (see also [14]).

The ^1^H, and ^13^C-NMR spectra of the 16.8 min fraction showed this to be a mixture of two components, both aromatic in nature. The major component gave ^1^H-NMR signals at δ 6.96 (d, *J* = 8.3 Hz), δ 7.5 (d,d *J* = 1.9, 8.3 Hz) and δ 7.73 (d, *J* = 1.9 Hz). A combination of HSQC and HMBC spectra gave ^13^*C*-H signals at δ 121.1, 126.6 and 117.4 and non-protonated ^13^C signals at δ 134.5, 148.9, 153.7 and 174.8. Similarly the minor component gave ^1^H signals at δ 6.65 (d, *J* = 8.3 Hz), δ 7.02 (d, *J* = 1.8 Hz) and δ 7.22 (d,d *J* = 1.8, 8.3 Hz), with corresponding ^13^C shifts measured from the HSQC spectrum at δ 117.7, 117.3 and 125.8. There was insufficient signal/noise in the HMBC spectrum to give information on the non-protonated ^13^C shifts. However these data were not sufficient to assign structures.

The ^1^H- and ^13^C-NMR spectra of the 28.5 min fraction indicated a structure more complex than any of the above. An accurate *m/z* value of 294.1503 was measured; the molecular formula C_19_H_20_NO_2_ has calculated mass 294.1489, *i.e.*, Δ = 4.8 ppm. Consideration of all available 1-D and 2-D NMR data led to the candidate structure **3** (see Figure 1). This was identified as roemrefidine, which has been reported previously [15], and is the *N*-methyl derivative of the alkaloid roemerine.

The ^1^H and ^13^C chemical shifts determined here for roemrefidine are given in Table 1. The 3-bond (vicinal) ^1^H-^1^H coupling constants between the protons at positions 4 and 5, and positions 6 and 7 fall into two ranges; 3.9 to 5.8 Hz, and 13.0 to 14.0 Hz. The smaller coupling range is typical for C-H bond dihedral angles *ca*. 60° or *ca*. 120°, whereas the larger coupling constants are typical for dihedral angles nearer 180°.

**Table 1:** ^1^H- and ^13^C-NMR data for roemrefidine (3) ^a^.

Position	δ ^1^H	δ ^13^C ^b^
1		143.3 ^c^
2		147.6 ^c^
3	6.82 (s)	106.8
3a		122.6
4 (Ψe)	3.05 (d,d *J* = 4.5, 18.0 Hz)	23.1
4 (Ψa)	3.32 (d,d,d *J* = 5.8, 13.0, 18.0 Hz)	
5 (Ψa)	3.67 (d,t *J* = 4.5, 13.0 Hz)	61.2
5 (Ψe)	3.76 (d,d *J* = 5.8, 13.0 Hz)	
6 (Ψa)	4.64 (d,d *J* = 3.9, 14.0 Hz)	68.2
7 (Ψa)	3.09 (t *J* = 14.0 Hz)	28.1
7 (Ψe)	3.44 (d,d *J* = 3.9, 14.0 Hz)	
7a		130.5
8	7.43 (d *J* = 7.4 Hz)	128.0
9	7.39 (t *J* = 7.4 Hz)	128.4
10	7.43 (t *J* = 7.4 Hz)	128.0
11	8.12 (d *J* = 7.4 Hz)	126.2
11a		130.4
11b		115.3
11c		118.5
OCH_2_O	6.07 (d *J* = 0.8 Hz)	101.3
	6.20 (d *J* = 0.8 Hz)	
CH_3_ (Ψa)	3.05 (s)	42.4
(Ψe)	3.38 (s)	53.0

^a^ Solution in CD_3_CN-D_2_O (1:1); ^b^ Measured from HSQC and HMBC spectra, and values are ± 0.3 δ; ^c^ These two assignments may be interchanged.

**Figure 2:**
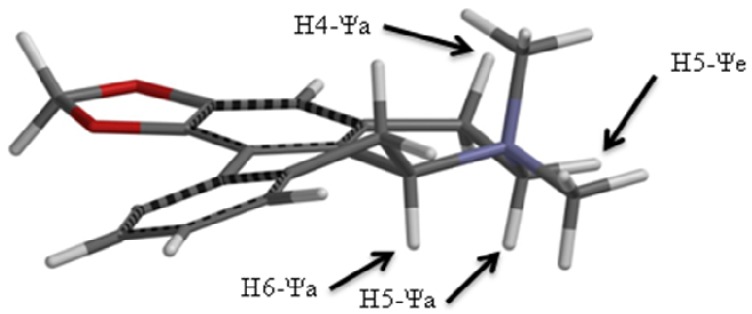
*Spartan* energy minimized structure of roemrefidine.

Accordingly an energy minimized structure for roemrefidine was generated (see section 3.8) and this is shown in Figure 2. Relative to the average plane of the molecule it is clear that the protons at positions 4,5,6 and 7 can be assigned as pseudo-axial (Ψa) or pseudo-equatorial (Ψe), and on this basis the smaller vicinal coupling constants are assigned to Ψe/Ψe interactions or to Ψa/Ψe interactions, and the larger vicinal coupling constants to Ψa/Ψa interactions.

The methyl signals were assigned as CH_3_ (Ψa) and CH_3_ (Ψe) since the NOESY spectrum showed a strong correlation between the CH_3_ signal at 3.38 δ and 4.64 δ (H6 Ψa), but only a very weak correlation between 3.05 δ and 4.64 δ. This indicates the methyl group giving the signal at 3.38 δ is closer in space to the H6 (Ψa) proton, which is the case for the Ψe methyl group (see Figure 2).

## 3. Experimental

### 3.1. Plant Material

Samples of the leaves of *Albertisia delagoensis* were collected in Tembe Elephant Park, Sihangwane [2732 AB], South Africa, in June 2005 and a voucher specimen [De Wet and SJ Siebert 100 (ZULU)] was deposited in the herbarium of the University of Zululand for verification purposes.

### 3.2. Extraction

The leaves were air-dried and powdered in a mill. Powdered leaf sample (10 g) was added to distilled water (100 mL), heated under reflux for one hour, cooled, filtered and lyophilised, yielding a brown powder (876 mg).

### 3.3. HPLC-DAD-on-flow-NMR

The hyphenated HPLC-DAD-NMR system consisted of an Agilent 1100 HPLC equipped with a column oven, a Bruker u.v. diode array detector (DAD) in the range 200 to 700 nm, a Bruker 36 place capillary loop storage device (BPSU36), and a Bruker DRX 600 NMR spectrometer with 3 mm cryogenic flow probe. The system was controlled with the Bruker *HyStar2.1* software.

HPLC conditions: A Thermo Hypersil (DBS) C_18_ column (5 μm, 150 × 4.6 mm) with an oven temperature 305 K. The gradient elution used D_2_O and CD_3_CN both containing 0.05% DCOOD, started with 98:2 (v/v) D_2_O-CD_3_CN 98:2, and linearly changed to 46:54 over 267 min, then was held at 46:54 for 33 min. Flow rate: 0.15 mL/min; Injection volume 100 μL; Sample concentration: a sample of the leaf extract (63 mg) was dissolved in D_2_O-CD_3_CN-DCOOD 98:2:0.05 (1 mL). The solution was filtered (0.45 μm filter with centrifugation at 10,000 g for 10 min), and the clear supernatant collected. DAD conditions: full range 200 to 700 nm. Alternative conditions used the same solvent composition but with the linear variation over 800 min, then held for 100 min. Flow rate: 0.05 mL/min.

### 3.4. HPLC-DAD-loop Storage

The HPLC-DAD, column and temperature, and DAD conditions were the same as used in the on-flow NMR experiments (see above).

HPLC conditions: Gradient elution used H_2_O and CH_3_CN both containing 0.1% HCOOH and the same solvent compositions were used as for the on-flow experiments. The linear variation of composition was over 40 min, then held for 5 min. Flow rate: 1 mL/min; Injection volume 20 μL; Sample concentration: as prepared for SPE (see above), but using normal (non-deuterated) solvents with 0.1% HCOOH.

The fractions for the specified peaks in the chromatogram were stored in separate loops. The experiment was repeated 6 times, and the corresponding fractions were combined. Each combined fraction was lyophilised, redissolved in D_2_O-CD_3_CN 1:1 (600 μL) containing 0.05% TSP (see below) and transferred to a 5 mm o.d. NMR tube for NMR measurements.

### 3.5. NMR Spectroscopy

Off-line NMR measurements used a Bruker DRX 600 spectrometer with a standard 5 mm ^1^H/^13^C inverse mode probe (^1^H-NMR at 600 MHz, ^13^C-NMR at 151 MHz). The residual ^1^H solvent signals from deuterated water and acetonitrile were suppressed using the WET method [16] with ^13^C decoupling. All spectra were measured at 292 K. The 2-D NMR experiments used were homonuclear ^1^H COSY, NOESY and TOCSY, and heteronuclear ^1^H-^13^C HSQC and HMBC, and these techniques have been summarised by Braun *et al*. [17].

### 3.6. Molecular Modelling

The software used for molecular modelling was *Spartan ’10* from Wavefunction Inc. (Irvine, CA, USA).

### 3.7. Mass Spectrometery

The accurate m/z value was measured by the King’s College, University of London, Mass Spectrometry service using a Bruker Apex III system with electrospray ionisation from methanol.

### 3.8. Allantoic Acid

An authentic sample of allantoic acid (**1**) was prepared by alkaline hydrolysis of allantoin, as described by Behrend and Schultz [18]. ^1^H-NMR (DMSO-d_6_) δ: 5.20 (1H, t, *J* = 7.9 Hz, C*H*), 5.77 (4H, br s, N*H*_2_), 6.78 (2H, d, *J* = 7.9 Hz, N*H*). ^13^C-NMR (DMSO-d_6_) δ: 57.8 (*C*H), 157.9 (*C*ONH_2_), 171.3 (*C*O_2_H).

## 4. Conclusions

HPLC-NMR analysis of the neutral aqueous extract of the leaves of *Albertisia delagoensis* identified the compounds present including *proto*-quercitol, nicotinic acid, allantoic acid, 3,4-dihydroxybenzoic acid, phthalic acid, and the aporphine alkaloid derivative roemrefidine. The ^1^H- and ^13^C-NMR spectroscopic data for allantoic acid and roemrefidine are reported for the first time. In addition molecular modelling of the roemrefidine structure allowed stereospecific assignment of the ^1^H resonances by comparison with vicinal coupling constants measured from the ^1^H spectrum.

Munoz *et al*. [19] showed that roemrefidine, isolated from the stem bark of *Sparattanthelium amazonum* Martius (Hernandiaceae), is active against the malaria parasite *Plasmodium falciparumin vitro* and against *P. berghei* in mice, but showed no cytotoxic activity against the cell lines KB, HEp-2, and HeLa. It is *aqueous* extracts of the leaves of *A. delagoensis* that are reported [2] to be used in traditional medicine to treat fever. Since roemrefidine is a salt (quaternary nitrogen) it is more likely to be extracted into neutral aqueous solution than the less polar MOalkaloids [3,4,5,6,7,8], and therefore roemrefidine must be considered a candidate for the febrifugal activity of the traditional preparations. In addition we have recently demonstrated [20] the antimicrobial activity of roemrefidine towards *Bacillus cereus*, *Escherichia coli* and *Staphylococcus aureus*, which may be relevant to the use of *A. delagoensis* for the treatment of intestinal disorders.
